# Influence of Fungal Colonization on Exacerbations in Patients with Cystic Fibrosis

**DOI:** 10.3390/jof10120875

**Published:** 2024-12-17

**Authors:** Claudia Janeth Madrid-Carbajal, Teresa Peláez-García de la Rasilla, Marta Iscar-Urrutia, Marta Solís-García, Ramón Fernández-Álvarez, Liliana Pérez-Martínez, María Soledad Zapico-González, Marta Garcia-Clemente

**Affiliations:** 1 Pneumology Department, Central Universitary Hospital of Asturias (HUCA), 33011 Oviedo, Spain; marta.iscar@sespa.es (M.I.-U.); msolis1995@gmail.com (M.S.-G.); enelllano@gmail.com (R.F.-Á.); lila_jkr@hotmail.com (L.P.-M.); mgclemen@gmail.com (M.G.-C.); 2 Microbiology Department, Central Universitary Hospital of Asturias (HUCA), 33011 Oviedo, Spain; mtpelaez@gmail.com (T.P.-G.d.l.R.); marisolzapico@hotmail.com (M.S.Z.-G.)

**Keywords:** fibrosis cystic, aspergillosis, diagnostic, fungal screening, molecular techniques

## Abstract

The importance of fungal pathogens in cystic fibrosis (CF) patients and their diagnosis remains a challenge, so our aim was to analyze the influence of the detection of fungi in sputum by using conventional culture and molecular techniques, polymerase chain reaction (PCR), lateral flow devices (LFDs), and galactomannan (GM) on exacerbations in patients with cystic fibrosis. A prospective study was conducted in patients via follow-up in the CF Unit of the Central University Hospital of Asturias from January 2021 to April 2022. Adult patients with at least one documented exacerbation were included. A complete fungal analysis of sputum samples was performed both in a period of clinical stability and in the exacerbation period. The microbiological study included conventional cultures for fungi, qPCR (polymerase chain reaction), LFDs (lateral flow devices), and galactomannan (GM) in sputum. We found that there were changes in their detection according to whether the patient is in a period of clinical stability or exacerbation; the positivity of the molecular tests and biomarkers in the period of exacerbation increased by 14%, 25%, and 21% for the analysis by qPCR, GM, and LFDs for *Aspergillus* and by 15% for the sputum culture for *Aspergillus*, which may mean that fungal isolates may play a role in the exacerbations of these patients.

## 1. Introduction

Cystic fibrosis (CF) is one of the most frequent genetic diseases in the Caucasian race, with an incidence in Spain of approximately 1/5000 live births [1,2]. It is a pathology with autosomal recessive inheritance, so it is estimated that between 4 and 5% of Caucasians are carriers of the disease. It is a complex and progressive disease that affects the secretion of exocrine glands at the levels of different organs and systems. The hydroelectrolytic and mucoprotein alterations in these glands cause thick and abnormal secretions that produce obstruction and favor infection.

Chronic bronchopulmonary infection by bacterial pathogens is well known and has been widely studied in CF patients, resulting in an inflammation–obstruction infection process that leads, in turn, to te morphologic and functional deterioration of the lung with the development of bronchiectasis [3]. In addition to chronic bacterial bronchial infection, CF patients are predisposed to fungal colonization because of the ability of some fungi to colonize the lower respiratory tract and because of the frequent courses of antibiotics that these patients receive in order to achieve good control of their disease.

The role of fungi isolated in the respiratory secretions of CF patients is undefined, and the role they may play in respiratory exacerbations is even more unknown [4].

The role of fungal detection in respiratory exacerbations is increasingly being studied, and current studies have shown that *Aspergillus* and *Pseudallescheria*/*Scedosporiumare* are the fungi most frequently found in pulmonary exacerbations in these patients, usually manifesting as the disease progresses [5,6]. Other rare fungi, such as *Exophiala dermatitidis*, *Lomentospora prolificans,* and *Rasamsonia argillacae*, have also been associated with exacerbations in patients with cystic fibrosis [7,8].

*Aspergillus fumigatus* has been the most frequently isolated fungus in the sputum of CF patients, with 35.3% of samples being positive for this fungus in the study by Guegan et al. [9]. However, although culture-based detection methods are the main approach for fungal isolation, processing guidelines are not standardized and it is possible to improve the performance of fungal pathogen detection when using other molecular tests [10]. PCR offers higher sensitivity, increasing fungal detection by more than 40%, according to some studies [9,11]. The determination of galactomannan (GM), which is a polysaccharide of *Aspergillus* species, also contributes to improved diagnosis. In the study by Nuh A et al., the usefulness of GM sputum was retrospectively validated [12], concluding that this test is useful in the diagnosis of chronic pulmonary aspergillosis and allergic bronchopulmonary aspergillosis, showing adequate concordance with clinical diagnosis or sputum culture. In any case, in all of these molecular tests, the interpretation of the results must be performed according to the clinical context.

The risk factors for the detection of *Aspergillus* spp. in patients with CF are not entirely clear due to the heterogeneous methodology and sample size of the different studies, but repeated courses of antibiotics, inhaled corticosteroids, impaired pulmonary function, frequent exacerbations, co-infection with other potentially pathogenic microorganisms, and even CFTR mutations are suggested.

About 2000 CFTR mutations have been identified, of which the single amino acid deletion F508del is the most common and accounts for about 70% of the disease.

Several studies have highlighted that the type of pathogenic CFTR variant may influence disease manifestations and the colonization of the lungs by respiratory pathogens. However, the relationship between the CFTR genotype and the clinical spectrum of *A. fumigatus* in CF has not yet been fully elucidated [13].

The importance of fungal pathogens in CF patients and their diagnosis remains a challenge, so our aim was to analyze the influence of the detection of fungi in sputum by conventional cultures and molecular techniques (PCR, LFDs, and GM) on exacerbations in patients with cystic fibrosis for the early diagnosis and facilitate therapeutic decisions or predict functional worsening in CF patients.

## 2. Materials and Methods

A prospective study was conducted in patients under follow-up in the CF Unit of the Hospital Universitario Central de Asturias from January 2021 to April 2022. Adult patients, aged > 18 years, with at least one documented exacerbation during the study period were included. A complete fungal analysis of sputum samples was performed in all cases, both in the period of clinical stability and in the period of exacerbation. The microbiological study included conventional cultures for fungi, qPCR, LFDs (lateral flow devices), and GM in sputum, trying to determine the differences between both periods. For this purpose, two sputum samples were collected from each patient at two different times throughout the study, one in the stability phase and the other in the exacerbation, by spontaneous expectoration in a sterile container.

We obtained the patient’s clinical history and epidemiological, microbiological, and treatment data in a specific protocol for this study. Pulmonary function parameters were collected every three months and, in case of exacerbation, one month after the episode.

Chronic bronchial fungal colonization was defined when *Aspergillus fumigatus* was isolated in two or more sputum samples analyzed the year before the study [14].

We define respiratory exacerbation as the presence of changes in the usual respiratory signs and symptoms requiring treatment, with the presence of two or more of the following signs: changes in sputum volume and color, increased cough, fatigue or dyspnea, weight loss or anorexia, a decrease of 10% or more in lung function, or radiological changes [15].

We have considered severe pulmonary exacerbations as those that produce a drop of more than 10% of forced expiratory volume in 1 s (FEV1) with respect to the period of stability or that present hypoxemia or require hospital admission and/or the need for intravenous antibiotics [16].

For the microbiological analysis, the following methodology was used:

1. Fungal cultures from respiratory samples on Sabouraud dextrose agar plates (BioMerieux, Mercy, L’Etoile, France). Nonselective fungal medium (Sabouraud dextrose agar) (SDA) supplemented with 500 mg/L chloramphenicol. All plates were incubated for at least 10 days at 30 °C for four weeks.
2. Fungal identification was performed using a matrix-assisted laser desorption ionization–time-of-flight (MALDI-TOF) mass spectrometry instrument (Bruker, Spain), following the manufacturer’s instructions.
3. Both sealed culture media and sputum samples from the stability phase and sputum from exacerbations are sent to the microbiology service at room temperature no longer 2 h later.
4. The commercially manufactured LFD (AspLFD, OLM Diagnostics, Newcastle upon Tyne, UK) with a visual reader provides a semi-quantitative evaluation and eliminates subjectivity when interpreting the results.
5. Quantitative real-time PCR for the genus *Aspergillus* is performed as follows: DNA extraction for qPCR analysis was performed on an automated ELITe InGenius platform, as well as qPCR using *Aspergillus* spp. The target region was ribosomal18S DNA (r18S rDNA), and the human B globin gene was used as the internal standard. The fungal DNA copy number was expressed as copies/mL relative to an rDNA18s standard curve.
6. Sputum for DNA extraction was placed immediately at 4 °C after collection and processed within 24 h. Sputum samples were processed using 5 %N-acetyl-L-cysteine at a 1:1 ratio for liquefying and then subsequently plated undiluted on culture media
7. The GM test was performed using Platelia™ *Aspergillus* (Bio-Rad Laboratories, Madrid, Spain) with a cutoff value of ≥0.5 in serum and ≥1.0 in bronchoalveolar lavage (BAL) or ≥4 in tracheal aspirate, bronchial aspirate. In sputum samples given the absence of standardization, we used a cutoff point of 0.7, according to the work of Nuh A et al. [12].
8. Bacteriological culture sputum is sowed in plates with both general (Chocolate PVX Agar, Columbia blood agar +5% lamb blood) and selective (MacConkey MCK Agar, BCSA Agar, S.aureus SAIDE Chromogenic Agar, *S. aureus* MRSA Chromogenic Agar) solid culture media from Biomerieux. We incubated the culture for 72 h in 5% CO_2_ atmosphere at 37 °C, reading the growth daily. Isolated colonies of potentially pathogenic microorganisms are identified by MALDI-TOF according to the manufacturer’s instructions.

A group of patients have voluntarily collaborated in the evaluation of the environmental influence of fungi. Environmental samples were collected at the home of volunteer patients through spore collection by gravimetric means (Merck Air Sampler MAS100 NT portable). Plates were exposed for 48 h using a selective culture medium for fungi (Sabouraud dextrose agar with chloramphenicol) in two areas of the house (in the bathroom and the patient’s room). Samples were sealed by the patients themselves and sent to the microbiology service for analysis. Sealed Sabouraud dextrose-irradiated plates were incubated at 30 °C for 5 days. These were examined daily to check for fungal growth. Colonies suggestive of *A. fumigatus* were identified growing on the plates.

The project was authorized by the Research Ethics Committee of the Principality of Asturias (project number: 2021.080) and met the ethical conditions required. The patients were informed of the methodology and objectives of the study by means of an information sheet, and all of them signed informed consent to participate in the study.

A descriptive analysis was performed. Proportions or means with standard deviation (SD) described the patient sample. Continuous variables were compared using Student’s *t*-test, while chi-square or Fisher’s exact tests were used for the analysis of categorical dependent data, as appropriate. McNemar’s test was used to compare proportions for two related samples to compare two clinical moments (stability and exacerbation) of the same sample. All tests were bilateral, and a *p* value < 0.05 was considered statistically significant. Statistical analysis was performed using SPSS version 25.0.

## 3. Results

**Figure 1:**
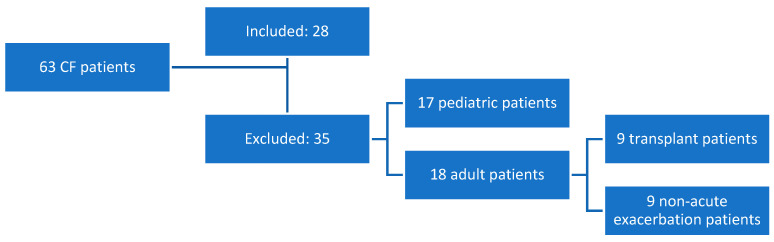
Flow chart.

From a total of 63 patients with a diagnosis of CF under follow-up in the Cystic Fibrosis Unit of Asturias, 17 patients of pediatric age were excluded. We also excluded nine patients who required lung transplantation because of the interference that immunosuppression can generate in the interpretation of the results. Finally, nine patients were excluded because they did not present any exacerbation during the study period (Figure 1).

### 3.1. Epidemiological and Clinical Data

A total of 28 patients were included, all of whom presented at least one respiratory exacerbation during the study period. The mean age was 32 years (20–52), with 16 patients (57%) being male. Regarding the genetic study, 14 patients (50%) were homozygous for the Phe508del mutation and 12 patients (43%) were heterozygous for the Phe508del/G542X mutation. Chronic bronchial colonization by *Aspergillus fumigatus* was observed in eight patients (29%). The general data of the sample are shown in Table S1.

### 3.2. Microbiological Findings

**Figure 2:**
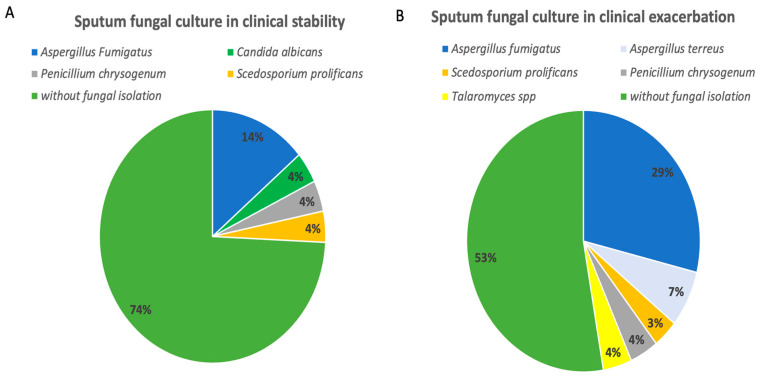
Mycological culture according to clinical condition. This figure shows how there is an increase in fungal detection by sputum culture according to the clinical period. (**A**) Fungal detection is observed in the stability period. (**B**) Fungal detection is observed in the period of clinical exacerbation.

An increase in fungi detection in the exacerbation period was observed in 21%. Figure 2 describes the fungi isolated according to the clinical period (stability or exacerbation), showing a clear increase in *Aspergillus* spp. in the exacerbation stage. When we analyze the species, *Aspergillus fumigatus* predominates both in the stability and exacerbation stages, increasing its detection by 15% in the exacerbation stage (see Figure 2).

When analyzing the sputum culture data, it was observed that, in the exacerbation period, fungal isolation occurred in thirteen patients, wherein *Aspergillus fumigatus* was detected in eight patients (62%), and in six of these patients (75%), *Aspergillus fumigatus* was detected only in the exacerbation period. In two patients (25%), *Aspergillus fumigatus* was detected in sputum culture in both clinical periods (stability and exacerbation).

In the exacerbation period, other fungi such as *Aspergillus terreus* (13%), *Penicillium chrysogenum* (8%), *Talaromyces* spp. (8%), and *Scedosporium prolifecans* (8%) were also detected.

Figure 2 describes in detail the fungi isolated from sputum cultures collected at the stages of clinical stability (A) and exacerbation (B).

When comparing the analysis of the detection of *Aspergillus* in the periods of stability and exacerbation (Table 1), significantly higher detection was observed during exacerbations for all detection techniques (qPCR, GM, LFD) and sputum culture for *Aspergillus fumigatus*; although, overall, only the detection of galactomannan for *Aspergillus* spp. was statistically significant.

If we analyze this in more detail, of the 28 patients who presented exacerbation during our study, 26 patients were analyzed with molecular detection tests and biomarkers for *Aspergillus* spp. performed in the period of stability and exacerbation, of which 16 patients (62%) presented the positivization of any of the rapid detection techniques for *Aspergillus* spp. (see Table S2).

In this subgroup of patients, we found an increase in the detection of *Aspergillus* spp. by qPCR and the LFD in 35% of patients and GM in 27% of patients.

Their characteristics are shown in Table 2 where it is observed that these patients are older and have worse pulmonary function both in the clinical stability phase and in the exacerbation, the latter observation being statistically significant (*p* = 0.04 and 0.007) compared to patients in whom positivity was not observed in any of the tests performed.

**Figure 3:**
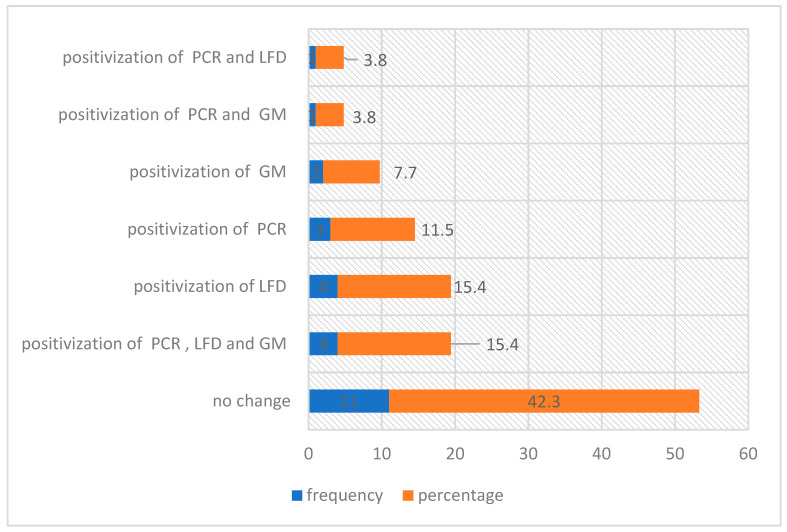
Distribution of changes in *Aspergillus* detection techniques in the exacerbation period of CF patients. qPCR: polymerase chain reaction, LFD: lateral flow device, GM: galactomannan.

Figure 3 shows the distribution of changes in fungal detection techniques according to the exacerbation of the patients analyzed; 57.6% of the cases presented some change in the molecular detection of *Aspergillus* during the period of clinical exacerbation.

The mean number of *Aspergillus* PCR copies in cases with positive sputum culture at the clinical stability stage was 27,687 (SD ± 32,258), and in cases with negative culture, this was 771 PCR copies (SD 594), the difference being statistically significant (*p* = 0.019).

The mean number of *Aspergillus* PCR copies in cases with positive sputum culture in the exacerbation stage was 13,747 (SD ± 27,561), and in cases with negative culture, this was 1020 PCR copies (SD ± 1422), with no significant difference (*p* = 0.246), see Table 3.

Throughout the study period, seven patients experienced severe exacerbation, observing in five patients (71.4%) positivity for qPCR, the LFD, and *Aspergillus* spp. galactomannan. Sputum culture was positive in three patients (43%).

Table 4 analyzes co-infection with *Pseudomonas aeruginosa* and *Staphylococcus aureus.* It observes that during the period of exacerbation and stability, the isolation of *Aspergillus* spp. was detected by qPCR predominates.

### 3.3. Treatment Data: Fungi Relationship with Inhaled Corticosteroids

Table 5 analyzes the relationship of treatment with inhaled corticosteroids. A higher proportion of positive tests for *Aspergillus* spp. qPCR in the stage of exacerbation of patients prescribed inhaled corticosteroids is observed, although without statistical significance.

### 3.4. Environmental Study of Fungi

**Figure 4:**
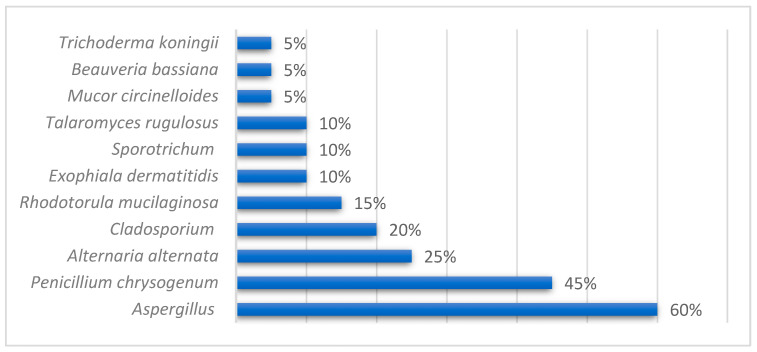
Environmental study of fungi in the bathrooms of CF patients.

**Figure 5:**
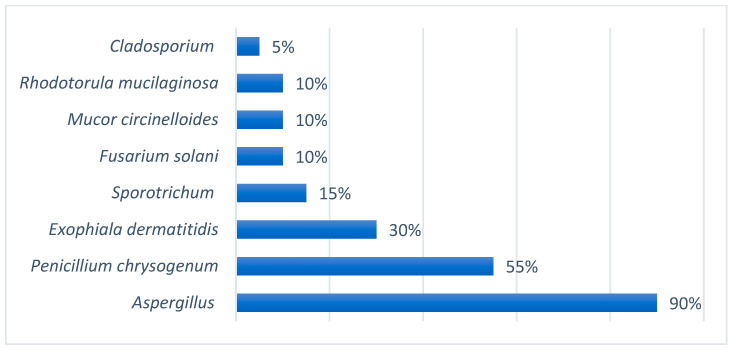
Environmental study of fungi in the rooms of CF patients.

Of the 28 patients analyzed, 20 were included in the environmental study of fungi. In the bathroom of these patients, *Aspergillus* spp. was identified predominantly in 60%, and in 45%, *Penicillium chrysogenumn* was identified. The rest is shown in Figure 4. When we analyzed the isolates in the rooms of the patients also undergoing the environmental study, *Aspergillus* spp. was identified in 90% and *Penicillium chrysogenumn* was identified in 55%; the rest is shown in Figure 5.

**Figure 6:**
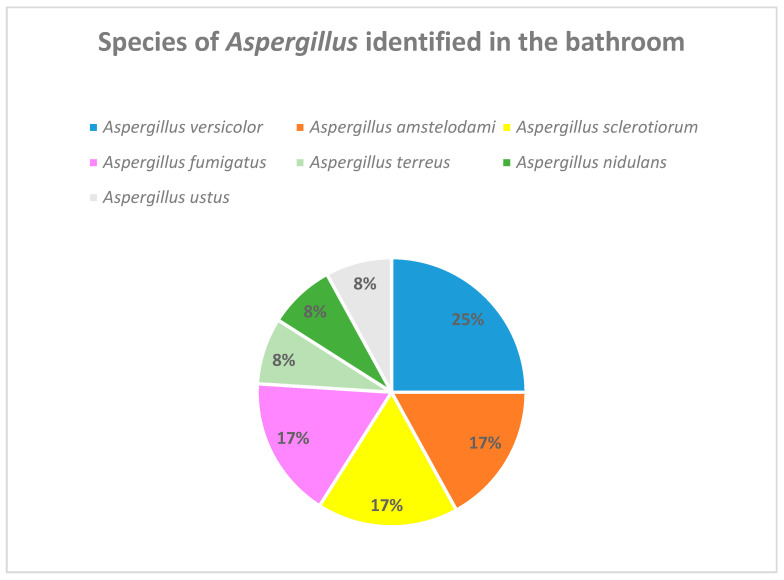
*Aspergillus* species identified in the bathrooms of the cystic fibrosis patients.

Figure 6 describes the *Aspergillus* species identified in the bathrooms of the cystic fibrosis patients who participated in the environmental study. *Aspergillus fumigatus* has been detected in 17% of the cultures in the bathroom of patients with cystic fibrosis.

**Figure 7:**
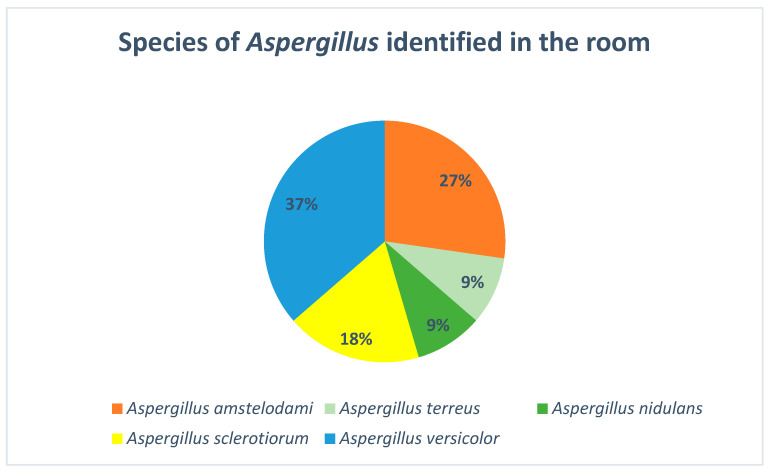
*Aspergillus* species identified in the rooms of the cystic fibrosis patients.

Figure 7 describes the *Aspergillus* species identified in the room of the cystic fibrosis patients who participated in the environmental study. *Aspergillus fumigatus* has not been detected in the environmental culture of the rooms.

## 4. Discussion

In recent decades, fungal colonization and respiratory infections have become increasingly important and have generated greater concern in patients with CF [8]. This has led to the search for better detection techniques that can improve the performance of sputum culture and allow early diagnosis. Until now, the impact that these new molecular techniques and biomarkers may have on the detection of fungi in the sputum of patients with CF has not been assessed, and the lack of standardization of these techniques complicates the evaluation of the pathogenicity of these microorganisms, making it difficult to assess their clinical relevance. In our study, in which we assessed the role of fungi during the period of clinical stability and in the exacerbations of patients with CF, we have observed, using conventional culture and molecular techniques, that there are changes in detection rates depending on whether the patient is in clinical stability or exacerbation. The positivity of molecular tests in the period of exacerbation increased by 14%, 25%, and 21%, depending on if the analysis was carried out by qPCR, GM, or the LFD for *Aspergillus* spp., and by 15% using sputum culture for *Aspergillus fumigatus*, which may mean that fungal isolates may play a role in the exacerbations of these patients.

It is noteworthy in our study that of the group of patients in whom any molecular detection technique and/or biomarkers for *Aspergillus* spp. was positivized in the exacerbation period, they were older patients and with worse pulmonary function both in the stable period and in the exacerbation period, identifying a group of patients in whom the role of colonization or infection by *Aspergillus* spp. should be established.

The clinical spectrum of pulmonary involvement by *Aspergillus* spp. is very broad, ranging from a hypersensitivity reaction (allergic bronchopulmonary aspergillosis) to an invasive disease with high mortality. Other less severe forms include chronic pulmonary aspergillosis, which usually occurs in immunocompetent patients with underlying pulmonary pathology [17].

When different microbiological techniques are evaluated, the conventional culturing of respiratory samples is still a useful detection method, but its cost-effectiveness is low [18] and other specific fungal detection methods need to be implemented in clinical practice. In the study by Baxter et al., *Aspergillus fumigatus* was the most frequently isolated fungus in sputum in patients with CF, with 35% of samples being positive, but the sensitivity of PCR was much higher, and more than 40% of the samples with negative culture had a positive *Aspergillus* PCR [11]. In our study, we also observed a higher sensitivity of qPCR in relation to sputum culture, obtaining 14% positivity in the stability period in sputum culture compared to 43% positivity in PCR, which means a detection of *Aspergillus* spp. in 29% more using PCR than sputum culture. The same was observed in the exacerbation period in which sputum culture was positive in 29% and PCR in 57%, which means a 28% higher detection of *Aspergillus* spp. by PCR compared to conventional culture. All this highlights the increased sensitivity of PCR in relation to culture, although the need for treatment must be assessed in the clinical context. In any case, although PCR is much more sensitive than culture, it is not known what the number of copies detected in the PCR of *Aspergillus* spp. is that can predict the presence of fungi in sputum culture. In our study, we found an average of 27,687 copies of *Aspergillus* spp. to be detected in sputum culture in the clinical stability period.

Regarding the influence that fungal pathogens may have on exacerbations in CF patients, there is not much literature on this subject. In our study, it is important to highlight that the conventional culture of *Aspergillus* spp. was 15% more positive in exacerbation in relation to the time of clinical stability, which may mean that in the exacerbation stage, fungi can increase their fungal load and this fungal load could have an impact on the severity of exacerbation.

However, it is not only the importance that fungal detection may have in the exacerbation period, but it is also important to assess the role that fungi may play in the progression of structural lung disease in patients with CF. Breuer O et al. [19]. in a study of 330 CF patients, concluded that fungal infections are associated with the progression of structural lung disease in young children with CF. This study highlights the need for further evaluation of early *Aspergillus* spp. infections and the feasibility, risk, and benefit of eradication regimens [19].

In this regard, molecular techniques and biomarkers may contribute to this early diagnosis, and the need for treatment should be assessed in the clinical context. In 2008, an immunochromatographic test known as the *Aspergillus* lateral flow device was described, which is specific for a 40 kDa glycoprotein secreted during the active growth of *Aspergillus* spp. Few studies in the scientific literature have analyzed the cost-effectiveness of LFDs in sputum from patients with chronic respiratory diseases. In the study by Wei Xiao et al., they concluded that sputum LFDs for the diagnosis of invasive pulmonary aspergillosis showed a sensitivity of 63% and a specificity of 91%. The sensitivity of the sputum LFD test is slightly lower than that described in BAL (about 80%) for the diagnosis of invasive pulmonary aspergillosis (IPA) in non-hematologic patients, but was still considerably higher than that of serum-based and fungal culture-based tests. Therefore, they conclude that sputum LFD testing has great potential for the early diagnosis of IPA [20]. In our study, the LFD detected 21% more positive results in the sputum sample during exacerbation than in the stable period, which may signify this active growth of the fungus during the period of exacerbation and thus its possible involvement in the pathogenesis of exacerbation.

Galactomannan (GM), a cell wall polysaccharide of *Aspergillus* spp., serum, or BAL antigen testing, is useful for the diagnosis of invasive pulmonary aspergillosis in high-risk populations, but the role of GM in the diagnosis of *Aspergillus* lung disease in the CF population is less clear [21]. Baxter et al. tested the *Aspergillus* enzyme immunoassay to detect GM in CF sputum from 146 adults with CF, and they found that 68 patients (48%) had detectable GM antigens [22]. In our study, 18% of positive samples were detected in the stable stage, while in the acute stage, positive samples were detected in 43%, similar to the aforementioned study, with this change being statistically significant according to the clinical stage referred to. Once again, therefore, the possible importance of fungal pathogens in the exacerbations of the disease becomes evident.

In an attempt to assess the possible sources of colonization, an environmental study was carried out, in which the presence of *Aspergillus* spp. was observed in 90% of the samples collected in the patients’ bedroom, and 60% positivity was found in the samples collected in the bathroom. Exposure to an environmental reservoir of *Aspergillus* represents an a priori risk factor for the colonization of the upper respiratory tract, leading to sensitization or even infection, and environmental measures should therefore be maximized in these patients [23].

Very few studies have been conducted on the environmental fungal exposure of cystic fibrosis patients, and, to our knowledge, there are no studies that address the relationship between specific fungal environmental exposure in the home and fungal colonization/infection. Establishing whether colonization by *Aspergillus* and other fungi is an environmental cause is something that should be investigated to provide primary prevention measures.

Epidemiological studies indicate that there is a wide variation in the prevalence of chronic co-infection with *Pseudomonas aeruginosa* and *Aspergillus fumigatus*, ranging from 16 to 35% in the Irish CF population, and a recent meta-analysis showed a prevalence of 15.8% with a significant variation, ranging from 2.3% to 44.8% among CF patients [24,25]. In our study, this co-infection was objectified in three patients (12%). *Aspergillus fumigatus* infection is found in many CF patients after *Pseudomonas aeruginosa* infection, and it is likely that *Pseudomonas aeruginosa* facilitates the establishment and growth of *Aspergillus fumigatus* [26].

As for potentially pathogenic microorganisms and *Aspergillus* co-infection, the predominant microorganism in the acute stage has been analyzed (Table 4). A predominance of positive PCR for *Aspergillus* spp. was observed in patients whose microorganism isolated in exacerbation was *Pseudomonas aeruginosa* in 80% (*p* = 0.17); there was no such predominance for the LFD and even less for GM.

In our study, we have evaluated the changes in fungal isolation by molecular methods, biomarkers, and sputum culture according to the clinical stage of the patient and its relationship with inhaled corticosteroids, without finding statistical significance; even a lower proportion of fungal isolation is observed in patients with inhaled corticosteroids independent of the clinical context. In this regard, Noni et al., in their retrospective cohort study of 121 CF patients born between 1988 and 1996, have concluded that the first isolation of *Aspergillus fumigatus* and chronic colonization are associated with the duration of treatment with inhaled corticosteroids [27]. Another study has identified inhaled and oral corticosteroids as risk factors for *Aspergillus* persistence [28]. Data on the relationship between inhaled corticosteroids and the development of *Aspergillus* spp. isolates have been mixed in previous studies. This possible relationship warrants further investigation, as inhaled corticosteroids are commonly prescribed for chronic maintenance therapy in the cystic fibrosis population, despite little data to support their use in the absence of asthma [29].

This study has highlighted the importance of fungal infection in CF patients and the need for early diagnosis in order to tailor treatment according to the clinical context. Finding a diagnostic algorithm with new serological and molecular tests in easily collected samples such as sputum would be the perfect method for the early detection of aspergillosis in susceptible patients.

Our study has an important limitation, which is the small sample size, justified in this case since 93% of the sample presented the Phe508del mutation, which makes them candidates for the initiation of triple CFTR modulating therapy at present, thus contributing to a lower sputum production in these patients and therefore to a lower possibility of fungal surveillance by simple collection methods such as sputum. In addition, these therapies cause fewer exacerbations and thus have limited the number of patients enrolled. It has been discussed that the CFTR genotype may correlate with infection and the pathogenicity of certain pathogens, potentially interfering with treatment with CFTR modulators. Some studies have reported a higher proportion of patients with the homozygous DF508del genotype in patients with *Aspergillus fumigatus* colonization [30]. Despite the sample size, this study opens the way for future research on the role of fungi in exacerbations in CF patients, especially in the new era of CFTR modulators. On the other hand, another of our limitations has been that not all patients have participated in the fungal environmental study. However, this work has the potential to investigate different clinical situations in order to analyze the impact of fungal screening on the disease.

It seems evident that fungal detection by new molecular techniques, biomarkers, and sputum culture is higher in the clinical exacerbation stage, which could raise the question of whether microbiological factors such as co-infection with other potentially pathogenic microorganisms, repeated courses of antibiotics and inhaled corticosteroids, or the severity of the disease could be determinants. We depend on the patient’s clinical manifestations and response to antibiotic treatment to be able to indicate antifungal treatment.

The value of the current study is to identify the importance of *Aspergillus* spp. in exacerbations in cystic fibrosis patients. The goal is not to assess the performance of the new CFTR protein-modulating therapies in chronic bronchial fungal colonization/infection; while it is true that exacerbations decrease with the advent of the new CFTR-modulating therapy, exacerbations still occur, and it is now even more important to elucidate an early diagnostic method in detecting fungus and determine if treatment is necessary. The issue of *Aspergillus* spp. decline in the era of new CFTR protein modulators is recently under development [31].

In our study, we observed that persistent fungal isolation may change depending on the clinical situation of the patient (whether the patient is stable or exacerbated). Further studies are needed to elucidate the clinical impact of *Aspergillus* spp. colonization in patients with cystic fibrosis and to define patients who would benefit from antifungal treatment to prevent lung function deterioration or lung damage [30]. An initial approach might involve trying to elucidate the difference between persistent colonization and *Aspergillus* spp. infection using current diagnostic methods. In addition, it is important to master the patient’s clinical status with therapeutic intervention.

## Figures and Tables

**Table 1:** Detection of *Aspergillus* according to clinical stage.

*Aspergillus* Detection Technique	Stability	Exacerbation	*p*
qPCR * positive for *Aspergillus*	12 (43%)	16 (57%)	0.267
Copies/mL (qPCR) *°	12,228 + 23,582	14,823 + 31,731	0.6
*Aspergillus* GM * positive	6 (18%)	12 (43%)	0.021
LFD * positive *Aspergillus*	7 (25%)	13 (46%)	0.065
*Aspergillus fumigatus* positive fungal cultures	4 (14%)	8 (29%)	0.219

* qPCR: polymerase chain reaction, LFD: lateral flow device, GM: galactomannan *p:* probability calculated with McNemar’s test. *° Copies/mL (qPCR), *p*: probability calculated by independent samples *t*-test unless otherwise indicated.

**Table 2:** Characteristics of patients with positivization of any *Aspergillus* spp. detection technique in exacerbation period.

Features	Positivization of Any *Aspergillus* Detection Techniquen (qPCR, LFD or GM *•)	N	Media	SD *	*p* *
Age (year)	yes	16	32.4	10.4	0.8
	no	10	31.3	7.5	
Number of exacerbations	yes	16	1.6	0.7	0.5
	no	10	1.4	0.4	
FEV1 * in percent at stability (%)	yes	16	58	16.5	0.04
	no	10	78	23.1	
Absolute FEV1 * (mL) at stability	yes	16	2038	793	0.012
	no	10	2963	916	
FEV1 * in percentage in exacerbation	yes	16	50	17.4	0.007
	no	10	76	23.9	
Absolute FEV1 * (mL) in acute exacerbation	yes	16	1747	757	0.02
	no	10	2803	744	

*• qPCR: polymerase chain reaction, LFD: lateral flow device, GM: galactomannan. * FEV1: forced expiratory volume in 1 s * SD: standard deviation * *p*: probability calculated by independent samples *t*-test unless otherwise indicated.

**Table 3:** Relationship of *Aspergillus* qPCR copies with *Aspergillus fumigatus* culture.

qPCR (Number of Copies)	Positive Culture	Negative Culture	*p*
Stability period	27,687 ± 32,258	771 ± 594	0.019
Period of exacerbation	13,747 ± 27,561	1020 ± 1422	0.246

qPCR: polymerase chain reaction, *p*: probability calculated by independent samples *t*-test unless otherwise indicated.

**Table 4:** Potentially pathogenic microorganisms (PPMs) in the acute stage in relation to fungal detection techniques.

	*Pseudomonas aeruginosa*	*p*	*Staphylococcus aureus*	*p*
*Aspergillus fumigatus* culture			*Aspergillus fumigatus* culture	
Positive	4 (50%)	0.26	4 (46%)	0.64
Negative	4 (50%)		9 (69%)	
qPCR *Aspergillus*			qPCR *Aspergillus*	
Positive	8 (80%)	0.17	7 (54%)	0.27
Negative	2 (20%)		6 (43%)	
LFD *Aspergillus*			LFD *Aspergillus*	
Positive	5 (55%)	0.92	6 (50%)	0.68
Negative	4 (45%)		6 (50%)	
GM *Aspergillus*			GM *Aspergillus*	
Positive	4 (40%)	0.74	4 (31%)	0.165
Negative	6 (60%)		9 (69%)	

qPCR: polymerase chain reaction, LFD: lateral flow device, GM: galactomannan. *p*: probability calculated with Chi-square test.

**Table 5:** *Aspergillus fumigatus* detection in patients using inhaled corticosteroids (ICs) in relation to periods of stability and exacerbation.

Stability	Patients with IC * N: 18	%	*p* *	Exacerbation	Patients with IC * N: 17	%	*p* *
Positive LFD *	5	28%	0.6	Positive LFD	8	47%	0.3
Negative LFD*	13	72%		Negative LFD	9	53%	
GM positive *	3	17%	0.8	GM positive	6	35%	0.1
GM negative *	15	83%		GM negative	11	65%	
qPCR positive *	7	39%	0.6	qPCR positive	10	59%	0.7
qPCR negative *	11	61%		qPCR negative	7	41%	
Positive culture	3	17%	0.2	Positive culture	8	47%	0.9
Negative culture	15	83%		Negative culture	9	53%	

* qPCR: polymerase chain reaction, LFD: lateral flow device, GM: galactomannan. * IC: inhaled corticosteroids. * *p*: probability calculated with Chi-square test.

## Data Availability

Data is contained within the article.

## Supplementary Materials

The following supporting information can be downloaded at: https://www.mdpi.com/article/10.3390/jof10120875/s1, Table S1: General data of the sample; Table S2: Distribution of comorbidity of patients with cystic fibrosis and number of exacerbations, admissions and severe exacerbations. Table S3: Detail of bacterial, fungal culture and molecular detection techniques in stability and exacerbation of cystic fibrosis patients.

## Abbreviations

ABPA	Allergic bronchopulmonary aspergillosis
BAL	Bronchoalveolar lavage
CF	Cystic fibrosis
FEV1	Forced expiratory volume in the first second
GM	Galactomannan
GERD	Gastroesophageal reflux disease
IC	Inhaled corticosteroids
IPA	Invasive pulmonary aspergillosis
LFD	Lateral flow device
PCR	Polymerase chain reaction
PPM	Potentially pathogenic microorganisms
MALDI-TOF	Matrix-assisted laser desorption ionization–time-of-light
SD	Standard deviation
