# Supplementary material for: Influence of Fungal Colonization on Exacerbations in Patients with Cystic Fibrosis
Source: J Fungi (Basel). 2024 Dec 17;10(12):875. doi: 10.3390/jof10120875 (PMC11676277; doi:10.3390/jof10120875)
Supplement: Supplementary file 1 [file jof-10-00875-s001.zip › jof-3308970-supplementary.pdf]

Table S1. General data of the sample.

**GENDER**	NO.	%
Male sex	16	57%
Female sex	12	43%
**GENETICS**		
Homozygosis Phe508del	14	50%
Heterozygosis Phe508del/G542X	12	43%
Other mutations	2	7%
**PULMONARY COMORBIDITIES**		
Asthma	14	50%
ABPA	4	14%
**OTHER COMORBIDITIES**	21	75%
Osteoporosis	8	29%
Chronic sinusitis	8	29%
Diabetes associated with CF	6	21%
Gastroesophageal reflux disease (GERD)	4	14%
Hepatopathy	4	14%
**LUNG FUNCTION (forced expiratory volume in 1 second: FEV1)**		
FEV1>80%	9	32%
FEV1 50-79%	10	34%
FEV1 30-49%	9	32%
FEV1<30%.	0	0%
**NUMBER OF EXACERBATIONS**		
1 exacerbation	18	64%
2 exacerbations	7	25%
3 or more exacerbations	3	11%
Exacerbation with admission	4	14%
Severe exacerbation	7	25%
**CHRONIC TREATMENT**		
Inhaled antibiotics	20	71%
Inhaled corticosteroids	18	64%

Table S2. Distribution of comorbidity of patients with cystic fibrosis and number of exacerbations, admissions and severe exacerbations.

Patient	Comorbidity	Exacerbation (number)	Income (number)	Exacerbation Severe (due to decreased pulmonary function)
1	CFRD*<br>Osteoporosis<br>Chronic sinusitis<br>Pancreatic insufficiency	2	0	0
2	Osteoporosis<br>Pancreatic insufficiency	2	0	yes
3	CFRD* CFRD* CFRD* CFRD* CFRD* CFRD* CFRD<br>Osteoporosis<br>Pancreatic insufficiency	3 o >	1	yes
4	Hepatopathy<br>Pancreatic insufficiency	2	0	no
5	CFRD* CFRD* CFRD* CFRD* CFRD* CFRD* CFRD<br>Pancreatic insufficiency	1	0	no
6	Pancreatic insufficiency<br>Hepatopathy	2	0	yes
7	Chronic sinusitis<br>Pancreatic insufficiency	1	0	no
8	Pancreatic insufficiency	3 o >	0	no
9	Chronic sinusitis<br>Pancreatic insufficiency	2	0	no
10	Pancreatic insufficiency<br>Hepatopathy	1	0	no
11	Pancreatic insufficiency<br>Hepatopathy	1	0	no

12	Osteoporosis<br>GERD **<br>Pancreatic insufficiency<br>Hepatopathy	3 o >	1	yes
13	Pancreatic insufficiency	1	0	no
14	Pancreatic insufficiency	2	0	no
15	Pancreatic insufficiency	1	0	no
16	No comorbidity	1	0	yes
17	Chronic sinusitis<br>Pancreatic insufficiency	1	0	no
18	Osteoporosis<br>Pancreatic insufficiency	1	0	no
19	Osteoporosis<br>Pancreatic insufficiency	1	0	no
20	CFRD*<br>Osteoporosis<br>Chronic sinusitis	1	1	no
21	Osteoporosis<br>Chronic sinusitis	1	1	yes
22	DRCF *<br>Chronic sinusitis<br>Pancreatic insufficiency	1	0	Yes
23	CFRD *<br>GERD **<br>Osteoporosis<br>Pancreatic insufficiency	1	0	no
24	GERD **<br>Chronic sinusitis	1	0	no
25	Pancreatic insufficiency	1	0	no
26	Pancreatic insufficiency<br>Hepatopathy	2	0	no

27	GERD **<br>Hepatopathy<br>Pancreatic insufficiency	1	0	no
28	GERD **<br>Hepatopathy	1	0	no

*CFRD: Diabetes related to cystic fibrosis**GERD: gastroesophageal reflux disease

Table S3. Detail of bacterial, fungal culture and molecular detection techniques in stability and exacerbation of cystic fibrosis patients.

	**Cultivation and molecular techniques**	**Stability test date**	**Acute testing date**	**PPM at stability**	**PPM in aggravation**
**1**	Bacterial	13/4/21	9/2/21	Pseudomonas aeruginosa<br>Staphylococcus aureus	Pseudomonas aeruginosa<br>Staphylococcus aureus
	Fungal	13/4/21	9/2/21	*Scedosporium prolificans*	*Scedosporium prolificans*
	PCR *Aspergillus*	13/4/21	9/2/21	negative	negative
	GM *Aspergillus*	13/4/21	9/2/21	negative	negative
	LFD *Aspergillus*	13/4/21	9/2/21	negative	negative
**2**	Bacterial	12/2/21	23/3/21	*Pseudomonas aeruginosa*	*Pseudomonas aeruginosa*<br>*Stenotophomonas maltophilia*
	Fungal	12/2/21	23/3/21	No insulation	No insulation
	PCR *Aspergillus*	12/2/21	23/3/21	positive	positive
	GM *Aspergillus*	12/2/21	23/3/21	positive	positive
	LFD *Aspergillus*	12/2/21	23/3/21	negative	positive
**3**	Bacterial	4/3/21	12/1/21	*Staphylococcus aureus*	*Staphylococcus aureus*
	Fungal	4/3/21	12/1/21	No insulation	*Aspergillus fumigatus*
	PCR *Aspergillus*	4/3/21	12/1/21	negative	positive

	GM *Aspergillus*	4/3/21	12/1/21	negative	positive
	LFD *Aspergillus*	4/3/21	12/1/21	Negative	positive
**4**	Bacterial	11/5/21	24/2/21	*Pseudomonas aeruginosa*<br>*Staphylococcus aureus*	*Pseudomonas aeruginosa*<br>*Staphylococcus aureus*
	Fungal	11/5/21	24/2/21	*Penicillium chrysogenum*	*Aspergillus fumigatus*
	PCR *Aspergillus*	11/5/21	24/2/21	positive	positive
	GM *Aspergillus*	11/5/21	24/2/21	negative	negative
	LFD *Aspergillus*	11/5/21	24/2/21	negative	positive
**5**	Bacterial	16/2/21	11/5/21	*Pseudomonas aeruginosa*	*Pseudomonas aeruginosa*
	Fungal	16/2/21	11/5/21	No insulation	No insulation
	PCR *Aspergillus*	16/2/21	11/5/21	negative	positive
	GM *Aspergillus*	16/2/21	11/5/21	negative	negative
	LFD *Aspergillus*	16/2/21	11/5/21	negative	negative
**6**	Bacterial	16/2/21	26/1/21	*Staphylococcus aureus*	*Staphylococcus aureus*<br>*Achromobacter xylosoxidans*
	Fungal	16/2/21	26/1/21	No insulation	No insulation
	PCR *Aspergillus*	16/2/21	26/1/21	negative	negative
	GM *Aspergillus*	16/2/21	26/1/21	negative	positive

	LFD *Aspergillus*	16/2/21	26/1/21	negative	negative
**7**	Bacterial	12/1/21	20/6/21	*Staphylococcus aureus* *Burkholderia cepacia*	*Burkholderia cepacia*
	Fungal	12/1/21	20/6/21	No insulation	No insulation
	PCR *Aspergillus*	12/1/21	20/6/21	negative	negative
	GM *Aspergillus*	12/1/21	20/6/21	positive	negative
	LFD *Aspergillus*	12/1/21	20/6/21	negative	positive
**8**	Bacterial	2/3/21	2/2/21	*Staphylococcus aureus* *Achromobacter xylosoxidans*	*Staphylococcus aureus*
	Fungal	2/3/21	2/2/21	No insulation	No insulation
	PCR *Aspergillus*	2/3/21	2/2/21	negative	negative
	GM *Aspergillus*	2/3/21	2/2/21	negative	negative
	LFD *Aspergillus*	2/3/21	2/2/21	negative	positive
**9**	Bacterial	12/1/21	1/12/21	Normal bacterial flora	*Staphylococcus aureus*
	Fungal	12/1/21	1/12/21	*Aspergillus fumigatus*	*Aspergillus terreus*
	PCR *Aspergillus*	12/1/21	1/12/21	positive	positive
	GM *Aspergillus*	12/1/21	1/12/21	positive	positive
	LFD *Aspergillus*	12/1/21	1/12/21	positive	positive
**10**	Bacterial	20/7/21	22/6/21	*Staphylococcus aureus*	*Staphylococcus aureus*

	Fungal	20/7/21	22/6/21	No insulation	No insulation
	PCR *Aspergillus*	20/7/21	22/6/21	positive	positive
	GM *Aspergillus*	20/7/21	22/6/21	negative	negative
	LFD *Aspergillus*	20/7/21	22/6/21	positive	negative
**11**	Bacterial	29/6/21	2/8/21	*Staphylococcus aureus* *Pseudomonas aeruginosa*	*Staphylococcus aureus*
	Fungal	29/6/21	2/8/21	No insulation	No insulation
	PCR *Aspergillus*	29/6/21	2/8/21	positive	positive
	GM *Aspergillus*	29/6/21	2/8/21	negative	negative
	LFD *Aspergillus*	29/6/21	2/8/21	positive	positive
**12**	Bacterial	22/6/21	7/3/21	*Achromobacter xylosoxidans*	*Achromobacter xylosoxidans* *Staphylococcus aureus*
	Fungal	22/6/21	7/3/21	No insulation	No insulation
	PCR *Aspergillus*	22/6/21	7/3/21	positive	negative
	GM *Aspergillus*	22/6/21	7/3/21	negative	negative
	LFD *Aspergillus*	22/6/21	7/3/21	negative	negative
13	Bacterial	9/2/21	6/4/21	*Staphylococcus aureus*	*Staphylococcus aureus*
	Fungal	9/2/21	6/4/21	No insulation	No insulation
	PCR *Aspergillus*	9/2/21	6/4/21	negative	positive

	GM *Aspergillus*	9/2/21	6/4/21	negative	negative
	LFD *Aspergillus*	9/2/21	6/4/21	negative	negative
14	Bacterial	16/2/21	13/4/21	*Staphylococcus aureus* *Pseudomonas aeruginosa*	*Staphylococcus aureus* *Pseudomonas aeruginosa*
	Fungal	16/2/21	13/4/21	No insulation	*Aspergillus terreus*
	PCR *Aspergillus*	16/2/21	13/4/21	negative	positive
	GM *Aspergillus*	16/2/21	13/4/21	negative	positive
	LFD *Aspergillus*	16/2/21	13/4/21	negative	positive
**15**	Bacterial	28/9/21	05/5/21	*Staphylococcus aureus*	*Staphylococcus aureus*
	Fungal	28/9/21	05/5/21	No insulation	No insulation
	PCR *Aspergillus*	28/9/21	05/5/21	positive	negative
	GM *Aspergillus*	28/9/21	05/5/21	negative	negative
	LFD *Aspergillus*	28/9/21	05/5/21	negative	negative
**16**	Bacterial	13/4/21	20/7/21	*Staphylococcus aureus*	*Serratia liquefaciens*
	Fungal	13/4/21	20/7/21	*Aspergillus fumigatus*	No insulation
	PCR *Aspergillus*	13/4/21	20/7/21	negative	positive
	GM *Aspergillus*	13/4/21	20/7/21	positive	positive

	LFD *Aspergillus*	13/4/21	20/7/21	negative	positive
**17**	Bacterial	13/8/21	18/2/22	*Pseudomonas aeruginosa*	*Pseudomonas aeruginosa*
	Fungal	13/8/21	18/2/22	*Aspergillus fumigatus*	*Aspergillus fumigatus*
	PCR *Aspergillus*	13/8/21	18/2/22	negative	positive
	GM *Aspergillus*	13/8/21	18/2/22	negative	positive
	LFD *Aspergillus*	13/8/21	18/2/22	negative	positive
**18**	Bacterial	1/2/22	28/9/21	*Burkholderia cepacia*	*Burkholderia cepacia*
	Fungal	1/2/22	28/9/21	No insulation	No insulation
	PCR *Aspergillus*	1/2/22	28/9/21	positive	negative
	GM *Aspergillus*	1/2/22	28/9/21	negative	positive
	LFD *Aspergillus*	1/2/22	28/9/21	negative	negative
**19**	Bacterial	9/2/21	28/9/21	*Staphylococcus aureus* *Burkholderia cepacia*	*Burkholderia cepacia*
	Fungal	9/2/21	28/9/21	*Aspergillus fumigatus*	*Aspergillus fumigatus*
	PCR *Aspergillus*	9/2/21	28/9/21	positive	positive
	GM *Aspergillus*	9/2/21	28/9/21	positive	positive
	LFD *Aspergillus*	9/2/21	28/9/21	positive	positive
**20**	Bacterial	25/2/22	14/04/21	*Pseudomonas aeruginosa*	*Pseudomonas aeruginosa*
	Fungal	25/2/22	14/04/21	No insulation	*Aspergillus fumigatus*

	PCR *Aspergillus*	25/2/22	14/04/21	negative	negative
	GM *Aspergillus*	25/2/22	14/04/21	negative	negative
	LFD *Aspergillus*	25/2/22	14/04/21	negative	negative
**21**	Bacterial	13/12/21	6/4/22	*Cupriavidus metallidurans*	*Pseudomonas aeruginosa*
	Fungal	13/12/21	6/4/22	No insulation	*Talaromyces* spp
	PCR *Aspergillus*	13/12/21	6/4/22	negative	positive
	GM *Aspergillus*	13/12/21	6/4/22	negative	negative
	LFD *Aspergillus*	13/12/21	6/4/22	positive	positive
**22**	Bacterial	3/5/22	23/11/21	Normal bacterial flora	*Enterobacter cloacae*
	Fungal	3/5/22	23/11/21	No insulation	*Aspergillus fumigatus*
	PCR *Aspergillus*	3/5/22	23/11/21	negative	positive
	GM *Aspergillus*	3/5/22	23/11/21	negative	positive
	LFD *Aspergillus*	3/5/22	23/11/21	negative	positive
**23**	Bacterial	2/2/23	3/1/23	*Staphylococcus aureus*	*Pseudomonas aeruginosa* *Staphylococcus aureus*
	Fungal	2/2/23	3/1/23	No insulation	*Aspergillus fumigatus*
	PCR *Aspergillus*	2/2/23	3/1/23	negative	Not performed
	GM *Aspergillus*	2/2/23	3/1/23	positive	Not performed

	LFD *Aspergillus*	2/2/23	3/1/23	negative	Not performed
**24**	Bacterial	2/11/22	5/4/22	*Staphylococcus aureus*	*Staphylococcus aureus*
	Fungal	2/11/22	5/4/22	No insulation	No insulation
	PCR *Aspergillus*	2/11/22	5/4/22	negative	negative
	GM *Aspergillus*	2/11/22	5/4/22	negative	negative
	LFD *Aspergillus*	2/11/22	5/4/22	negative	negative
**25**	Bacterial	23/9/21	8/3/22	*Staphylococcus aureus*	*Pseudomonas aeruginosa*
	Fungal	23/9/21	8/3/22	No insulation	No insulation
	PCR *Aspergillus*	23/9/21	8/3/22	positive	negative
	GM *Aspergillus*	23/9/21	8/3/22	negative	positive
	LFD *Aspergillus*	23/9/21	8/3/22	negative	negative
**26**	Bacterial	14/12/21	22/3/22	*Pseudomonas aeruginosa*	*Pseudomonas aeruginosa*
	Fungal	14/12/21	22/3/22	*Candida albicans*	*Aspergillus fumigatus*
	PCR *Aspergillus*	14/12/21	22/3/22	negative	positive
	GM *Aspergillus*	14/12/21	22/3/22	negative	positive
	LFD *Aspergillus*	14/12/21	22/3/22	positive	negative
**27**	Bacterial	4/3/22	26/5/21	*Staphylococcus aureus*	*Staphylococcus aureus*
	Fungal	4/3/22	26/5/21	No insulation	*Penicillium chrysogenum*
	PCR *Aspergillus*	4/3/22	26/5/21	positive	positive

	GM *Aspergillus*	4/3/22	26/5/21	negative	negative
	LFD *Aspergillus*	4/3/22	26/5/21	negative	negative
**28**	Bacterial	9/8/21	9/10/21	*Proteus mirabillis*	*Pseudomonas aeruginosa*
	Fungal	9/8/21	9/10/21	No insulation	No insulation
	PCR *Aspergillus*	9/8/21	9/10/21	positive	Not performed
	GM *Aspergillus*	9/8/21	9/10/21	negative	Not performed
	LFD *Aspergillus*	9/8/21	9/10/21	positive	Not performed
